# Xylose induces cellulase production in *Thermoascus aurantiacus*

**DOI:** 10.1186/s13068-017-0965-z

**Published:** 2017-11-15

**Authors:** Timo Schuerg, Jan-Philip Prahl, Raphael Gabriel, Simon Harth, Firehiwot Tachea, Chyi-Shin Chen, Matthew Miller, Fabrice Masson, Qian He, Sarah Brown, Mona Mirshiaghi, Ling Liang, Lauren M. Tom, Deepti Tanjore, Ning Sun, Todd R. Pray, Steven W. Singer

**Affiliations:** 1 Biological Systems and Engineering Division, Lawrence Berkeley National Laboratory, 5885 Hollis Street, Emeryville, CA 94608 USA; 2 Institut für Genetik, Technische Universität Braunschweig, Braunschweig, Germany; 3 Advanced Biofuels Process Development Unit, Emeryville, CA USA

**Keywords:** *Thermoascus aurantiacus*, Xylose, Cellulases, Corn stover, Bioprocess, Thermophile, Filamentous fungi

## Abstract

**Background:**

Lignocellulosic biomass is an important resource for renewable production of biofuels and bioproducts. Enzymes that deconstruct this biomass are critical for the viability of biomass-based biofuel production processes. Current commercial enzyme mixtures have limited thermotolerance. Thermophilic fungi may provide enzyme mixtures with greater thermal stability leading to more robust processes. Understanding the induction of biomass-deconstructing enzymes in thermophilic fungi will provide the foundation for strategies to construct hyper-production strains.

**Results:**

Induction of cellulases using xylan was demonstrated during cultivation of the thermophilic fungus *Thermoascus aurantiacus*. Simulated fed-batch conditions with xylose induced comparable levels of cellulases. These fed-batch conditions were adapted to produce enzymes in 2 and 19 L bioreactors using xylose and xylose-rich hydrolysate from dilute acid pretreatment of corn stover. Enzymes from *T. aurantiacus* that were produced in the xylose-fed bioreactor demonstrated comparable performance in the saccharification of deacetylated, dilute acid-pretreated corn stover when compared to a commercial enzyme mixture at 50 °C. The *T. aurantiacus* enzymes retained this activity at of 60 °C while the commercial enzyme mixture was largely inactivated.

**Conclusions:**

Xylose induces both cellulase and xylanase production in *T. aurantiacus* and was used to produce enzymes at up to the 19 L bioreactor scale. The demonstration of induction by xylose-rich hydrolysate and saccharification of deacetylated, dilute acid-pretreated corn stover suggests a scenario to couple biomass pretreatment with onsite enzyme production in a biorefinery. This work further demonstrates the potential for *T. aurantiacus* as a thermophilic platform for cellulase development.

## Background

Lignocellulose present in plant biomass is an abundant resource for conversion to biofuels and other high-value chemicals and materials [1]. Lignocellulosic conversion processes rely on physical and chemical pretreatment and subsequent enzymatic hydrolysis to convert the biomass into sugar intermediates, which are then upgraded to fuels and chemicals. Cellulose, the major constituent of lignocellulosic biomass, is hydrolyzed by a mixture of enzymes that cleave different β-1,4-glycosidic bonds. Endoglucanases randomly hydrolyze bonds within the β-1,4-glucan chain while cellobiohydrolases hydrolyze cellulose from the reducing (type I) and non-reducing (type II) ends of the polymer releasing cellobiose. Beta-glucosidases subsequently hydrolyze cellobiose to glucose [2]. Lytic polysaccharide monooxygenases, which are recently discovered copper-dependent enzymes, complement the hydrolytic enzymes by oxidizing β-1,4-glycosidic bonds, increasing the overall efficiency of cellulose depolymerization [3–6].

High titer production of highly active and stable biomass-deconstructing enzymes still remains a challenge central to the conversion of biomass to biofuels [7, 8]. Mesophilic filamentous fungi, exemplified by *Trichoderma reesei*, are the most common platforms for industrial enzyme production that involve separate hydrolysis of pretreated biomass and fermentation [9]. These fungi produce enzymes which perform best at ~ 50 °C. Development of fungal platforms that produce enzymes that perform at higher temperatures and are more stable than current commercial enzyme mixtures will enable the use of high temperatures and shorter reaction times for saccharification, allowing utilization of waste heat, lowering viscosity at high solids loading and overcoming end-product inhibition [10]. Developing thermophilic fungi as platforms for enzyme production will provide a route to produce high temperature enzyme mixtures for biomass saccharification. The thermophilic filamentous fungus *Thermoascus aurantiacus* was found to be an intriguing host for enzyme production as it grows optimally at elevated temperatures (*T*_opt._ = 48–50 °C) while secreting large amounts of cellulases and hemicellulases that maintain high activity levels at temperatures up to 75 °C [11–13]. Individual *T. aurantiacus* glycoside hydrolases and lytic polysaccharide monooxygenases have been heterologously expressed in *T. ressei* [14], but development of *T. aurantiacus* as an alternative host will enable the production of new enzyme mixtures that can complement current commercial enzymes.

Understanding how cellulase and xylanase biosynthesis is induced in *T. aurantiacus* cultures is critical to establish this fungus as a thermophilic production platform. Production of extracellular enzymes by filamentous fungi is predominantly regulated transcriptionally and is mediated by low molecular weight sugars that are constituents of cellulose or hemicellulose [2, 15]. The action of these soluble inducers is counteracted by carbon catabolite repression (CCR), which ceases enzyme production when sugar concentrations become too high [2, 15, 16]. In *Aspergillus* species, particularly *A. niger*, expression of cellulases and hemicellulases is induced by xylose [17, 18]. In contrast, extensive studies on regulatory mechanisms of cellulase expression in *Neurospora crassa* have identified cellobiose as the primary inducer and suggested that xylose is the primary inducer for hemicellulases [19–21]. For *T. reesei*, a more complicated regulatory system has emerged and studies have demonstrated that both disaccharides (sophorose and lactose) as well as xylose are required for optimal induction of cellulases and hemicellulases. The combination of disaccharide and xylose as combined soluble inducers was exploited in a fed-batch process to produce high titers of cellulases and hemicellulases from *T. reesei* CL847, which is a hyper-production mutant [22].

Cellulase and xylanase production by *T. aurantiacus* has been performed in cultures with intact plant biomass and with purified components of biomass such as microcrystalline cellulose or xylan [12]. Hydrolyzed xylan has been used as inducer of cellulase and xylanase activities in *T. aurantiacus*, suggesting that both activities may be simultaneously induced by xylooligosaccharides [23]. Here we demonstrate that the *T. aurantiacus* cellulases and hemicellulases are strongly induced by xylose and xylose-induced cultivations can be performed at up to 19 L scale.

## Results

### Glycoside hydrolases are induced by xylan and Sigmacell cellulose

**Fig. 1:**
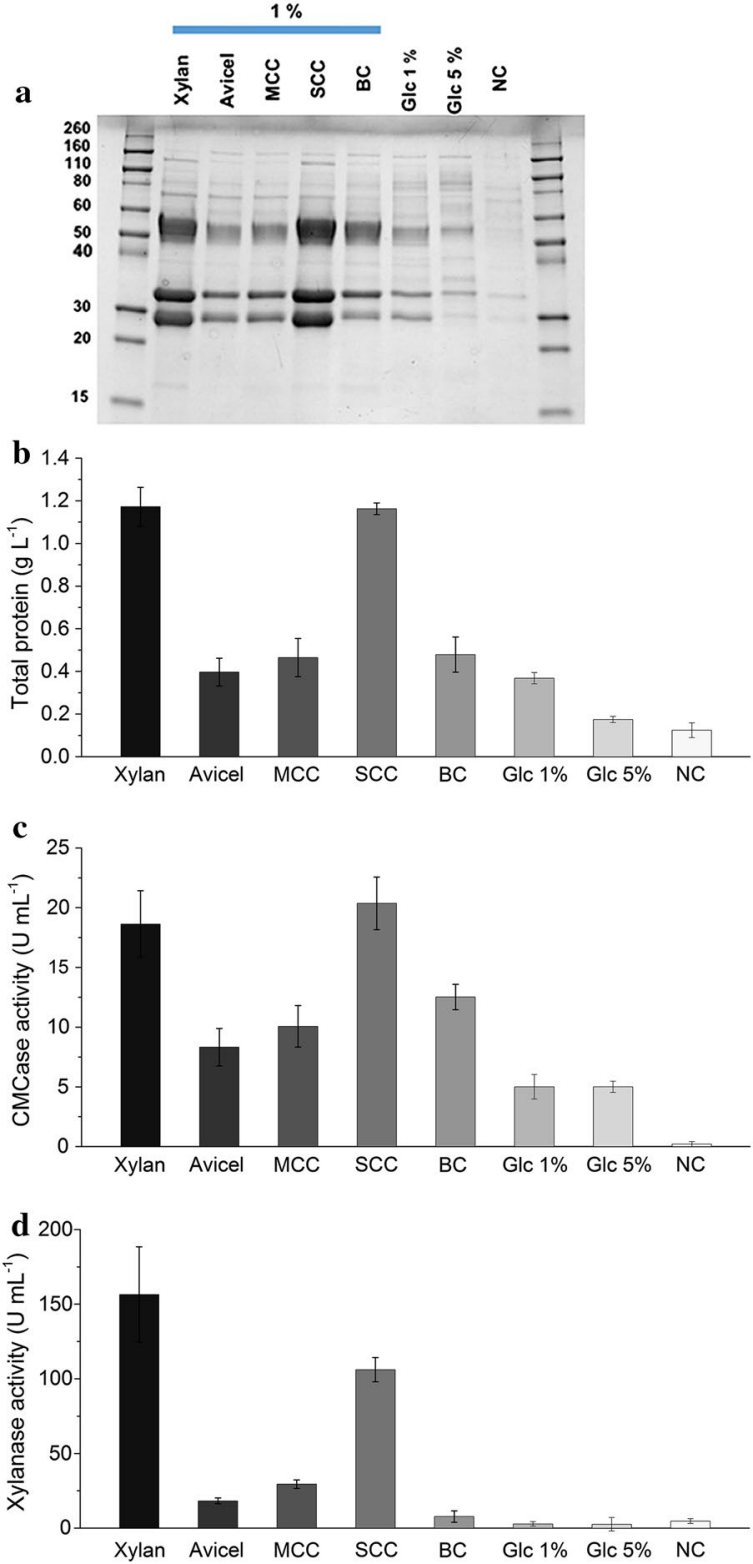
*T. aurantiacus* protein production with cellulose and xylan substrates. SDS-PAGE (**a**), protein concentration (**b**), CMCase activity (**c**), and xylanase activity (**d**) from supernatants of cultures recovered 72 h after shift of glucose-grown cultures to cellulose and xylan substrates. The cultures were pre-grown for 48 h in 2% glucose as carbon source and shifted to cultivation with 1% of each labeled carbon source. Cultivation of the mycelia after shifting to 1% glucose, 5% glucose and no carbon were used as controls. *MCC* micro crystalline cellulose, *SCC* Sigmacell cellulose, *BC* bacterial cellulose, *Glc* glucose, *NC* no carbon

To investigate glycoside hydrolase induction in *T. aurantiacus*, glucose-grown cultures were shifted to culture media containing purified hemicellulose (beechwood xylan) and cellulose substrates [Avicel, microcrystalline cellulose (MCC), Sigmacell cellulose (SCC), and bacterial cellulose (BC)] (Fig. 1a). Visualization of the supernatant proteins by SDS-PAGE demonstrated that the four major proteins previously produced from *T. aurantiacus* growing on pretreated switchgrass: GH7 (~ 54 kDa), GH5 (33 kDa), GH10 (33 kDa), and AA9 (25 kDa) were present at high levels in the xylan and Sigmacell cultures (Fig. 1b). Xylan and Sigmacell cellulose resulted in highest crude enzyme titers (> 1.1 g/L) and highest CMCase (> 19.5 U/mL) and xylanase (156.5 and 106.1 U/mL, respectively) activities. All other tested cellulose substrates (Avicel, MCC, and BC) demonstrated lower induction of glycoside hydrolases with crude enzyme titers < 0.5 g/L, CMCase activities < 12.7 U/mL, and xylanase activities < 29.5 U/mL. However, Avicel, MCC, and BC all had CMCases activities that were higher than glucose cultures and the Avicel and MCC cultures had higher xylanase activities than the glucose cultures (Fig. 1b–d).

### Xylose induces cellulase production in *T. aurantiacus*

**Fig. 2:**
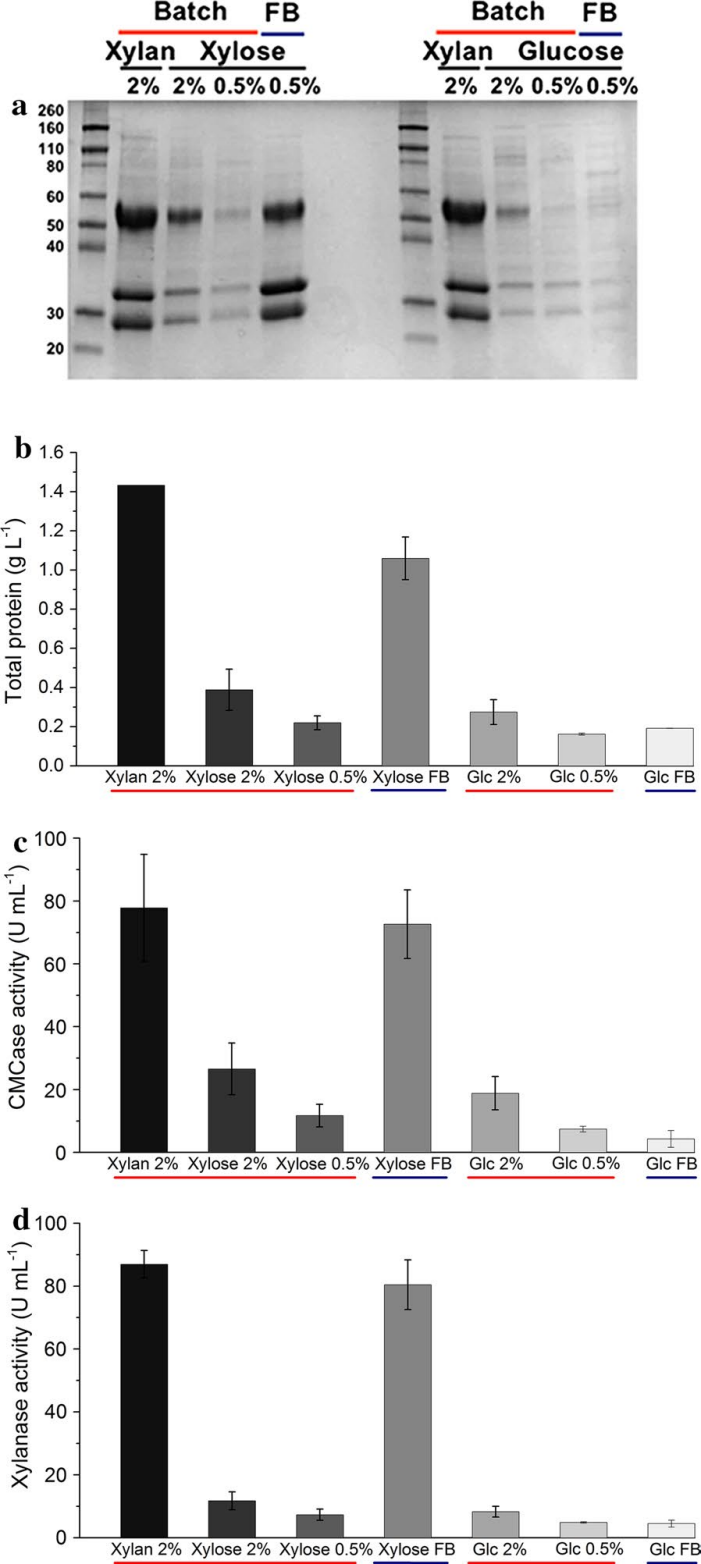
*T. aurantiacus* protein production with glucose and xylose. SDS-PAGE (**a**), protein concentration (**b**), CMCase activity (**c**), and xylanase activity (**d**) from supernatants of cultures recovered 72 h after shift of glucose-grown cultures to growth on glucose and xylose. Batch cultures were performed by adding glucose and xylose at the beginning of the cultivation and fed-batch cultures were performed by adding the sugars continuously using a peristaltic pump. Shift cultures with 2% beechwood xylan as the substrate were used as positive controls for protein production. Batch cultures are underlined in red and fed-batch cultures in blue

While the strong induction of the *T. aurantiacus* xylanase by beechwood xylan was not surprising, the strong induction of cellulases, as demonstrated by activity assays and SDS-PAGE, was an unexpected result. This observation suggested that xylose, continuously released at low levels during xylan cultivation, may induce *T. aurantiacus* to produce cellulases (GH7, GH5, AA9). To simulate continuous xylose release from xylan in a shake flask experiment, xylose was continuously fed at low quantities into *T. aurantiacus* shake flask cultivations using a peristaltic 12-channel low-flow pump. A continuous feed at 69.4 mg/L h d-xylose resulted in a 4.8-fold increase in protein production after 72 h compared to feeding the same amount of d-xylose in one pulse to a batch culture at the beginning of the cultivation (Fig. 2a, b). In the same comparison, CMCase activity was 6.2-fold higher and xylanase activity was 11-fold higher (Fig. 2c, d). A comparable glucose control feed did not result in significant protein production, confirming that the observed induction was specific for d-xylose.

### 2 L bioreactor fed-batch cultivations using xylose as inducer

**Fig. 3:**
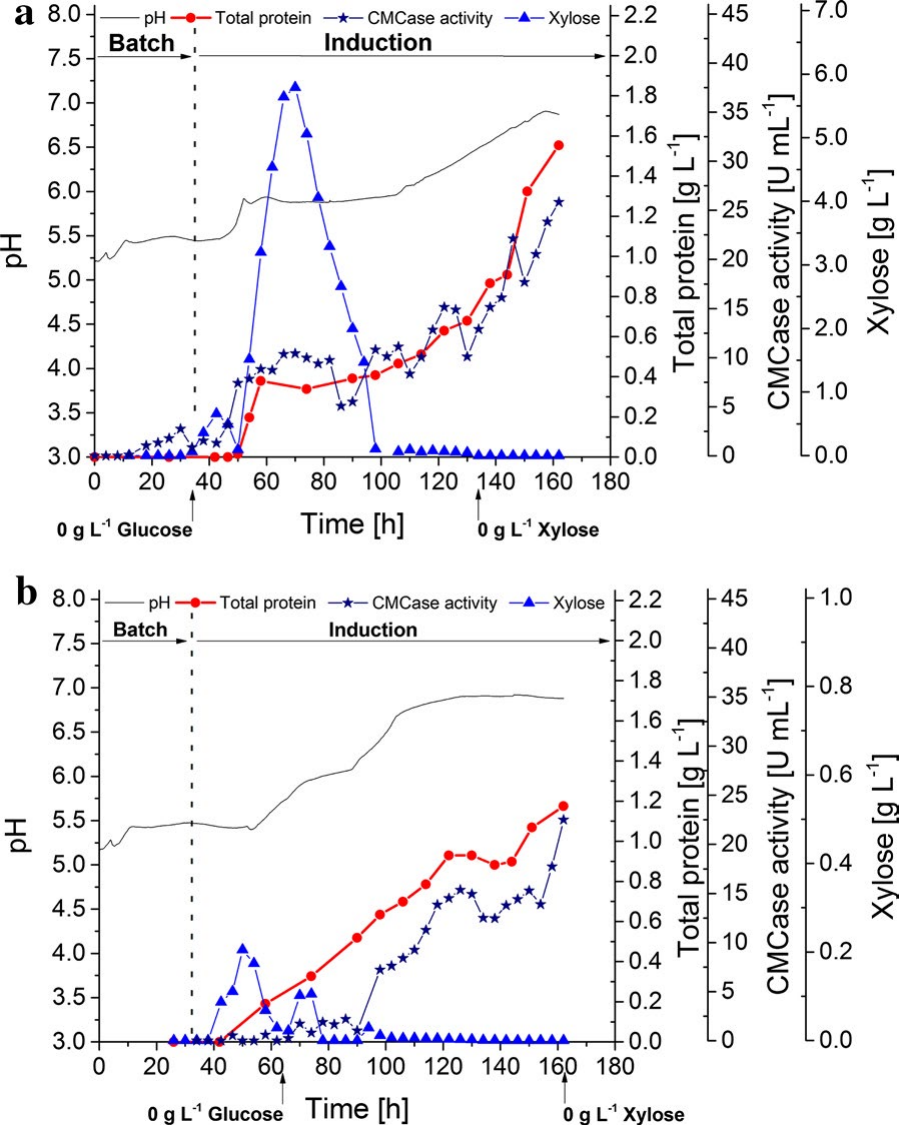
2 L bioreactor cultivation of *T. aurantiacus* under fed-batch conditions. *T. aurantiacus* protein production was performed using xylose (**a**) and xylose-rich hydrolysate (**b**) as substrate in fed-batch cultivations. The graph depicts pH (gray line), total protein (red circles), CMCase activity (blue stars), and xylose concentration (blue triangles) in the culture medium plotted against cultivation time

A 2 L fed-batch cultivation process for *T. aurantiacus* cellulase enzyme production was designed based on the xylose induction conducted in the simulated fed-batch mode (Fig. 3a). At a feed rate of 50.5 mg/L h d-xylose, a slight accumulation of d-xylose of up to 660 mg/L was observed within the first 12.5 h of feed. Shortly after, the accumulated xylose was consumed entirely, indicating that xylose metabolism increased while the feed rate was kept constant. Once a xylose concentration of 0 mg/L was measured, the protein titer increased sharply with a rate of 45.7 mg/L h. Ramping up the xylose feed at 51.2 h to 589.6 mg/L h resulted in a clear cessation of protein production and a strong accumulation of xylose up to 5.8 g/L. The xylose feed was stopped at 42.5 h, and a consumption rate of 184 mg/L h was detected. As soon as all xylose was consumed, a low xylose feed of 58.4 mg/L h, which was comparable to the initial feed, was started at 110 h. During the first 20 h after re-initiating the xylose feed, the protein titer increased only slightly with a rate of around 10.5 mg/L h until it started to increase strongly during the last 18 h of cultivation reaching a maximum productivity of 59.3 mg/L h. Increasing CMCase activity correlated with increasing protein titer, suggesting that the protein titer correlates with cellulase enzyme activities. The final protein titer and maximum CMCase activity reached 1.6 g/L and 25.8 U/mL after 162 h, respectively. An increase in pH was observed during the protein production phase, rising from an initial pH of 5.2–6.9, at which value the pH stabilized. A companion experiment was performed using a xylose-rich hydrolysate obtained using dilute acid-pretreated corn stover (Fig. 3b). The hydrolysate was fed at 113.2 mg/L h xylose and similar phenomena related to the pure xylose induction were observed, including: transient xylose accumulation, protein production after xylose consumption and pH rise related to protein production. A final titer of 1.2 g/L crude cellulase enzymes and CMCase activity of 22.5 U/mL was achieved from the xylose-rich hydrolysate.

### Impact of agitation and pH control

**Fig. 4:**
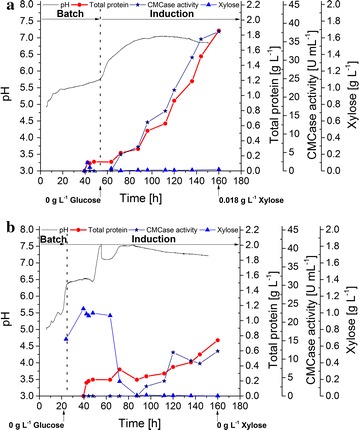
2 L bioreactor cultivation of *T. aurantiacus* at different agitation rates. *T. aurantiacus* protein production was performed at 200 rpm (**a**) and 400 rpm (**b**) using xylose as the substrate in fed-batch cultivations. The graph depicts pH (gray line), total protein (red circles), CMCase activity (blue stars) and xylose concentration (blue triangles) in the culture medium plotted against cultivation time

Based on the previous d-xylose fed-batch experiment, a low xylose feed of 58.4 mg/L h was determined to be optimal for cellulase enzyme production. Using this as a constant induction feed rate, constant stirring of 200 rpm vs. 400 rpm were compared (Fig. 4a, b). Glucose consumption during the batch phase was twice as high at 400 rpm vs. at 200 rpm (591.8 mg/L h vs. 224.4 mg/L h, respectively); however, d-xylose consumption was strongly reduced at 400 rpm, resulting in a significant accumulation of d-xylose (> 1 g/L) within the first 43 h of induction. A maximum productivity of 41.2 mg/L h and a final crude enzyme titer of 1.9 g/L was achieved when stirring at 200 rpm, while the maximum productivity and titer at 400 rpm were 16.0 mg/L h and 0.74 g/L, respectively.

**Fig. 5:**
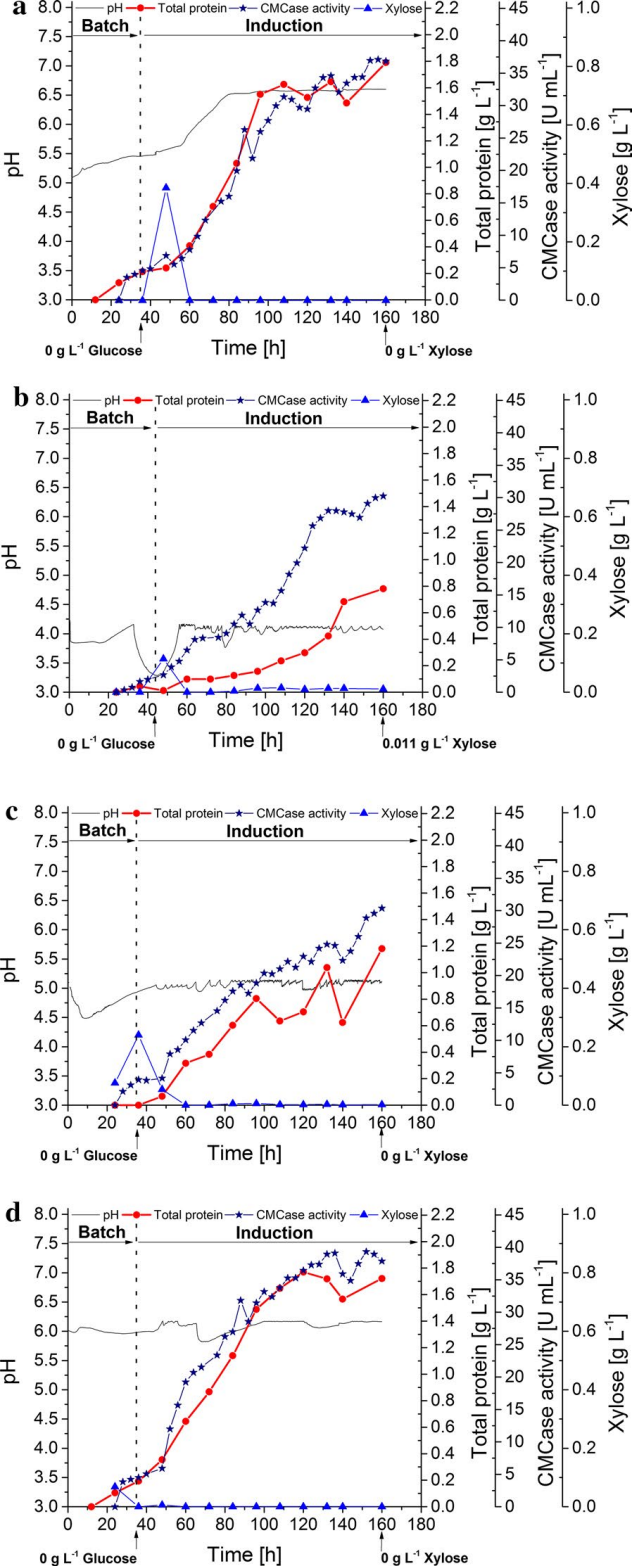
2 L bioreactor cultivation of *T. aurantiacus* at different pH values. *T. aurantiacus* protein production was performed with no pH control (**a**), at pH 4 (**b**), at pH 5 (**c**) and pH 6 (**d**) using xylose as the substrate in fed-batch cultivations. The pH was maintained by automated addition of HCl to cultures

In the xylose induction experiments described above, the initial pH was set to 5.0–5.2 and left uncontrolled, rising to ~ pH 7 during the protein production phase. The effect of pH in the *T. aurantiacus* cultivation was tested (Fig. 5a–d). Controlling the culture pH through automated addition of HCl to maintain pH at 6.0 was substantially beneficial compared to maintaining a controlled pH of 5.0 or 4.0, as the resulting maximal crude enzyme titers were 1.8, 1.2, and 0.8 g/L, respectively. The control experiment (initial pH 5.0, uncontrolled, final plateau at pH 6.6) resulted in a protein titer of 1.8 g/L, which was the same titer as for cultivation with the pH maintained at 6.0.

### Cultivation scale-up to 19 L bioreactor

**Fig. 6:**
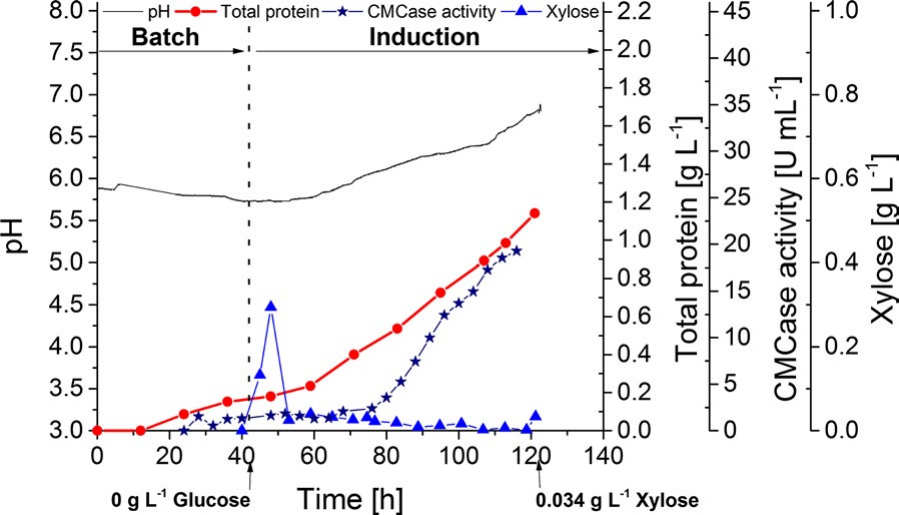
19 L bioreactor cultivation of *T. aurantiacus* under fed-batch conditions. *T. aurantiacus* protein production was performed using xylose as substrate in 19 L bioreactor cultivation. The graph depicts pH (gray line), total protein (red circles), CMCase activity (blue stars) and xylose concentration (blue triangles) in the culture medium plotted against cultivation time

Scaling up *T. aurantiacus* d-xylose-induced protein production to a 19 L bioreactor under uncontrolled pH conditions resulted in a maximum productivity of 19.5 mg/L h, a final crude enzyme titer of 1.1 g/L, and a maximum CMCase activity of 19.3 U/mL (Fig. 6). A transient accumulation of d-xylose up to 0.3 g/L was observed in accordance with previous 2 L fermentations, which may represent a metabolic adaptation from glucose to d-xylose consumption.

### Saccharification of pretreated corn stover using *T. aurantiacus* enzymes

**Fig. 7:**
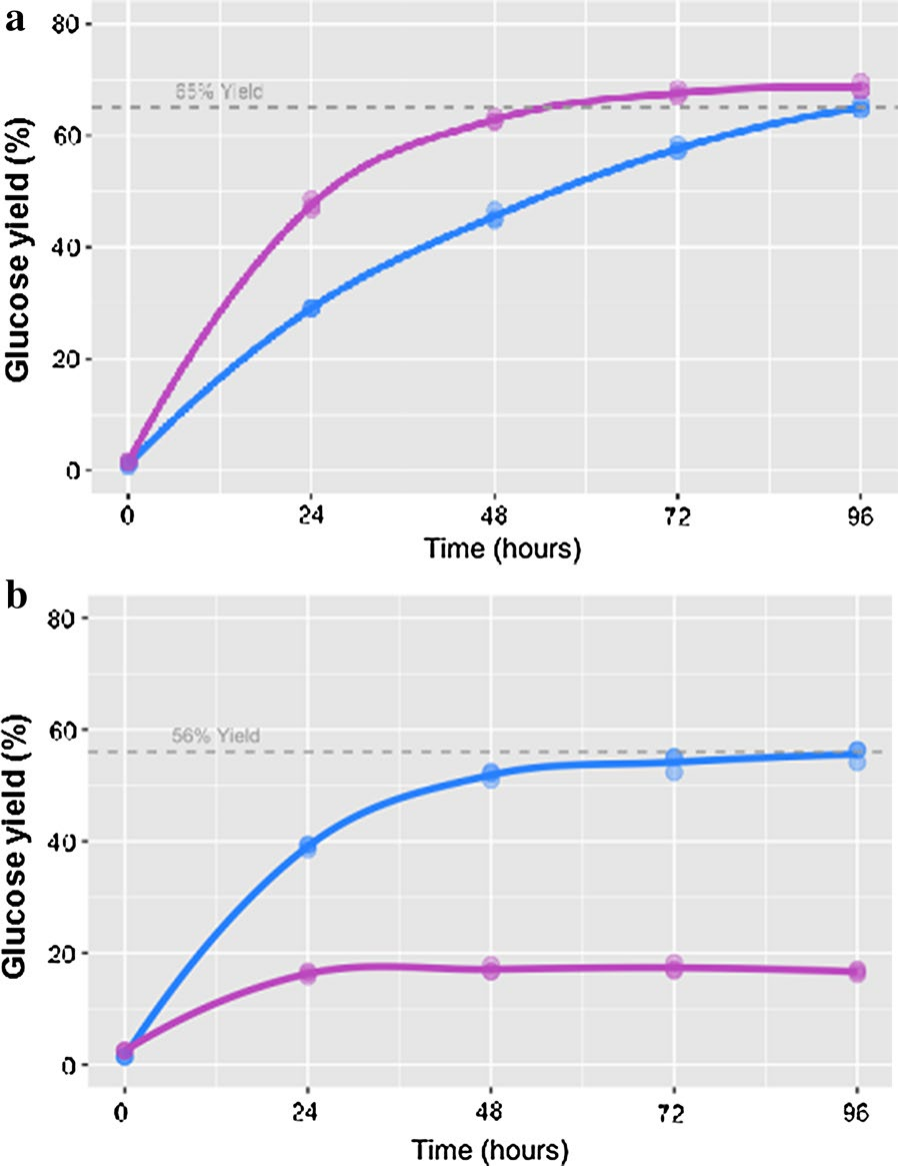
Saccharification of deacetylated, dilute acid-pretreated corn stover. Pretreated corn stover (2% w/v) was incubated at 50 °C (**a**) and 60 °C (**b**) with CTec2 and *T. aurantiacus* supernatant from xylose-induced cultures (20 mg/g glucan) for 96 h at pH 5 and glucose release measured by HPLC. Data points for *T. aurantiacus* are in blue and for CTec2 in purple. The dotted line depicts the saccharification yield from the *T. aurantiacus* enzymes

The supernatant from a 2 L bioreactor experiment, in which optimized d-xylose fed-batch conditions were used, was concentrated from 374 mL (1.85 g/L) to 73 mL (7.93 g/L) using tangential flow filtration (TFF). This protein concentrate was used to test the saccharification efficiency of the *T. aurantiacus* proteins in comparison to the commercially available enzyme cocktail CTec2 using pretreated corn stover. Saccharification was tested on deacetylated, dilute acid-pretreated corn stover. The experiments demonstrated that CTec2 and the *T. aurantiacus* proteins performed comparably in a glucose release assay at 50 °C (~ 70% glucose) (Fig. 7a). However, the *T. aurantiacus* proteins maintained their activity at 60 °C while the CTec2 enzymes appeared to be significantly deactivated (Fig. 7b).

## Discussion

Understanding the induction of fungal cellulase production by soluble sugars is an important requirement to scale cellulase production for the industrial conversion of biomass to biofuels and bioproducts. In this work, we have identified xylose as an inducer of both cellulases and xylanases in *T. aurantiacus* and have demonstrated its use in production of these extracellular enzymes at up to 19 L. Xylose induction of xylanases is commonly observed in filamentous fungi [24], and has previously been noted for *T. aurantiacus* [23], but xylose induction of both xylanases and cellulases has only been observed in *Aspergilli* (*A. niger* and *A. oryzae*), which are clustered phylogenetically with *T. aurantiacus* [25]. In *A. niger* and *A. oryzae*, the zinc finger transcription factor XlnR has been shown to regulate transcription of cellulase and xylanase genes, and *T. aurantiacus* possesses a XlnR gene that is likely the target for xylose in transcriptional activation of cellulase and xylanase genes [13].

The inductive effect of xylose was hypothesized based on batch cultivations of *T. aurantiacus* on purified beechwood xylan, which induced both cellulase and xylanase production. Batch cultivations on purified cellulose substrates produced variable levels of glycoside hydrolases that may be linked to the nature of these substrates. The Sigmacell cellulose cultures produced protein levels and glycoside hydrolase activities comparable to the xylan cultures; however the other two biomass-derived cellulose substrates, Avicel and microcrystalline cellulose, had lower levels of xylanase and CMCase activity. These activities were higher than the glucose-grown cultures, suggesting some level of induction from C6 soluble sugars produced by the cellulose substrates. This analysis is complicated by the presence of residual xylan in commercially available plant biomass-derived substrates [26]. The variations in xylanase and CMCase activity between Sigmacell, Avicel, and MCC may result from differential production of xylose during substrate consumption. To test this hypothesis, *T. aurantiacus* was cultured on bacterial cellulose (BC), which lacks the hemicellulose component. The BC—grown batch cultures had comparable CMCase activity to the Avicel and MCC cultures but negligible xylanase activity. This result suggests that there is some cellulase induction from C6 substrates, but that the xylose induction produces both cellulases and xylanases in *T. aurantiacus*.

The observation of xylose-induced production of *T. aurantiacus* cellulases enabled the scale-up of cultivation to 19 L using a fed-batch strategy that minimized carbon catabolite repression by overaccumulation of xylose in the culture medium. A similar strategy was employed with *T. ressei* CL847 to optimize protein production using a mixture of lactose and xylose as inducers [22, 27]. In *T. ressei* CL847 cultures, protein production commenced when the residual sugar concentration approached zero, releasing catabolite repression. A related approach to fed-batch production of cellulases was pursued in *T. reesei* Rut-C30, in which fed-batch protein production was induced by in situ generation of disaccharide inducers (sophorose, gentiobiose) from a glucose medium [28]. Protein production by wild-type *T. aurantiacus* described in this work can be improved by genetic modifications that release catabolite repression and improve expression of cellulases, as has recently been demonstrated for *Penicillium oxalicum* and *Myceliophthora thermophila* [29, 30]. These genetic modifications will be used to improve protein production in the fed-batch conditions with xylose as growth substrate and inducer for protein production. Testing of bioreactor parameters suggested that low levels of agitation and near neutral pH conditions promote enzyme production by *T. aurantiacus*.

The induction of *T. aurantiacus* cellulase production by xylose led to the use of xylose-rich hydrolysate obtained from dilute acid pretreatment of corn stover as an inducer for *T. aurantiacus*. Despite the complexity of this substrate, the behavior of the protein production system with the xylose-rich hydrolysate at 2 L scale was comparable to the behavior of the cultivation with pure xylose. Therefore, the xylose-rich hydrolysate may be a low-cost substrate for growth and induction of cellulase production in *T. aurantiacus*. Furthermore, the ability of the *T. aurantiacus* cellulases from xylose-induced cultures to saccharify a significant fraction of the glucan from dilute acid-pretreated corn stover suggests a scenario to couple biomass pretreatment with onsite enzyme production in a biorefinery. In this scenario, a portion of the xylose-rich hydrolysate obtained by dilute acid pretreatment of biomass will be used to grow *T. aurantiacus* and induce cellulase production. These cellulases will be used to saccharify the remaining glucan-rich fraction from dilute acid pretreatment to produce glucose for conversion to fuels and chemicals. Both the ability to generate the substrate for cellulase production onsite and the generation of thermostable enzymes will lower the cost of the conversion of plant biomass.

## Conclusions

In this work, we have shown that xylose induces both cellulases and xylanases from *T. aurantiacus*, a thermophilic fungus with promise to be a thermophilic protein production platform for biomass-deconstructing enzymes. Xylose induction was used to produce proteins from *T. aurantiacus* in 2 and 19 L bioreactors, and pH values near neutral were shown to be favorable for increased protein production. Protein production was also performed with xylose-rich hydrolysate from dilute acid pretreatment of corn stover. Saccharification of dilute acid-pretreated corn stover was performed at elevated temperature (60 °C). Combining the cellulase induction by xylose-rich hydrolysate with the high temperature saccharification of dilute acid-pretreated biomass provides a new model for onsite enzyme production at a biorefinery that use acid pretreatment.

## Methods

### Materials

#### Chemicals

All chemicals were purchased from Sigma-Aldrich unless otherwise indicated. Bacterial cellulose was extracted from commercial *Nata de coco* (Tropics) as previously described [31].

#### Biomass substrates

Deacetylated, dilute acid-pretreated corn stover was prepared as previously described [32]. The xylose-rich hydrolysate from dilute acid pretreatment was obtained by mixing 400 g dry weight corn stover (Idaho National Laboratory, Idaho, USA) with dilute H_2_SO_4_ with acid loading: 1 g H_2_SO_4_ per 100 g biomass and 10% biomass loading in an acid resistant Parr reactor (Series 4555 Floor Stand Reactors, 10 L, Hastelloy; Parr Instrument Company, Illinois, USA). The pretreatment conditions were as follows: temperature, 160 °C; agitation, 50 rpm; time, 10 min. After pretreatment, the liquid phase (xylose-rich hydrolysate) was separated from the solid phase by centrifugation (Sorvall RC 12BP Plus centrifuge; Thermo Scientific, Massachusetts, USA), operating at 5000 rpm and room temperature for 20 min. The hydrolysate obtained under these conditions contained 6.6 g/L d-xylose and 1.2 g/L glucose as measured by HPLC.

#### Microorganism and strain preservation

All experiments carried out in this work were performed with *T. aurantiacus* ATCC 26904, which was obtained from American Type Cell Culture Collection. To maintain the fungus, the strain was incubated on potato dextrose agar (PDA) at 49 °C for 2 days, followed by incubation at 45 °C for another 4 days. Spores were harvested by addition of 5 mL purified water. The resulting spore solution was mixed with 40% glycerol (1:1), frozen in liquid nitrogen and stored at − 80 °C.

#### *T. aurantiacus* shift experiments using purified cellulose and hemicellulose substrates

*Thermoascus aurantiacus* was grown in a pre-culture medium that was modified from the previously reported formulation [12] by replacing the carbon source with 2% glucose (w/v) and the nitrogen source with 0.8% soy meal peptone (w/v) as well as adjusting the pH to 6. This new formulation is referred to as modified McClendon medium. The cultivations were performed at 300 mL volume in 1 L baffled shake flasks with foam stopper sealing by inoculating the medium with 10 agar plugs from a PDA culture plate that had been grown at 50 °C for 6 days. Pre-cultures were incubated in a rotary shaker at 180 rpm and 50 °C for 48 h. The cell suspension was filtered through a glass-fiber funnel attached to a vacuum pump and washed with modified McClendon medium to remove remaining sugars. After filtration, 2 g of the mycelia (wet weight) were weighed into individual baffled culture flasks containing 50 mL of medium with different carbon sources as indicated and sealed with foam stoppers. All carbon sources were autoclaved separately and added to the flasks except for the insoluble substrates, which were autoclaved in the medium. The shift experiments were incubated in a rotary shaker at 180 rpm and 50 °C for 72 h. After the end of incubation, the amount of evaporated volume was replenished to 50 mL with sterile water and aliquots of the supernatant were filtered for further analysis.

#### Simulated fed-batch induction of *T. aurantiacus* protein production

The low feed was performed with a BT100-1L Multi-channel Peristaltic Pump (Langer Instruments Corp., Boonton, NJ, USA). The pump was assembled and calibrated with plastic cranks to ensure equal flow rates of the 12 individual channels. The flow rate was adjusted to 3.75 µL/min. Shift culture flasks of *T. aurantiacus* were prepared as described above. The batch treatment flasks received the respective amount of glucose or xylose after autoclaving. The feed tubes were inserted into the shake flasks for fed-batch cultivations. The incubation of fed-batch and batch cultures were performed for 72 h at 180 rpm and 50 °C.

#### Fed-batch fermentations to produce *T. aurantiacus* proteins in 2 L bioreactors

For the seed train of the inoculations for the 2 L bioreactor experiments, *T. aurantiacus* was grown in pre-culture medium. The cultivation was performed at 50 mL volume in 250 mL baffled shake flasks with foam stoppers by inoculating the medium with five agar plugs (*d* = 0.8 cm) obtained from a PDA culture plate which was grown at 50 °C for 6 days. Pre-cultures were incubated in a rotary shaker at 180 rpm and 50 °C for 48 h.

Two separate benchtop bioreactor systems, BIOSTAT^®^ B (Sartorius AG., Goettingen, Germany) and RALF Plus (Bioengineering Inc., Wald, Switzerland), were used at the 2 L scale to optimize the protein production process. The Sartorius BIOSTAT reactors are jacketed 2 L borosilicate glass vessels (UniVessel^®^, Sartorius AG, Goettingen, Germany) equipped with 2 × 6-blade disk impellers (Rushton impeller), a pH probe (Hamilton EasyFerm Plus VP 225, Bonaduz, Switzerland), and a dissolved oxygen (DO) probe (Hamilton VisiFerm DO 225, Bonaduz, Switzerland). The process parameters tested in these fermenters were as follows: an initial batch of 0.75 L was inoculated with 50 mL seed and incubated at 50 °C with an agitation at 200 rpm and air flow varying between 0.375 and 1.125 LPM (in batch phase) and 1.7 and 2.26 LPM (in production phase). Different feed solutions (medium B, medium C) were administered throughout the fed-batch phase of fermentation to each of the four reactors. Process values were monitored and recorded using the integrated Sartorius software (BioPAT MFCS/win). An autosampler (ASX-7100 Autosampler, Teledyne CETAC Technologies, Omaha, NE, USA) was connected to all four bioreactors and pre-programed to automatically take samples and store them at 4 °C. Bioengineering RALF reactors are jacketed 2 L glass vessels equipped with 2 × 6-blade disk impellers (Rushton impeller), a pH probe (Mettler Toledo Type 405-DPAS-SC-K8S/325 Pressurized gel-filled pH electrode, Mettler Toledo, Greifensee, Switzerland), and a DO probe (Mettler Toledo Oxygen Sensor InPro 6800 Gas, Mettler Toledo, Greifensee, Switzerland). The fermentation process parameters observed in these reactors were similar to those in Sartorius reactors, except agitation was varied between 200 and 600 rpm. During fed-batch phase in the campaign in Bioengineering RAFL reactors, 0.38 L of feed solution (medium B) was supplied to each of the two reactors. Process values were monitored and recorded using the integrated Bioengineering Inc. software (BioSCADA Lab).

#### Fed-batch fermentations to produce *T. aurantiacus* proteins in 19 L

The seed train for the 19 L bioreactor experiment was carried out at 300 mL volume in 1000 mL baffled shake flasks. Ten agar plugs were used for inoculation. After incubation, cell suspensions of different pre-culture batches were harvested and combined in a sterile container.

A 19 L Bioengineering NFL stainless steel reactor system (Bioengineering Inc., Wald, Switzerland) equipped with 2 × 6-blade disk impellers (Rushton impeller), a pH probe (Mettler Toledo InPro 3253I/SG/120, Mettler Toledo, Greifensee, Switzerland), and a DO probe (Mettler Toledo CH-8902 II 1/2G EX ia IIC SNCH 01, ATEX 3277 X, Mettler Toledo, Greifensee, Switzerland) was used to study scale-up of protein production. The process parameters tested in this study were based on the results from 2 L campaigns and were as follows: 14 L batch media, with an initial pH set to 5, was inoculated with 900 mL seed culture. A temperature of 50 °C was maintained with an agitation of 200 rpm and airflow of 15 LPM. Throughout the fermentation process, a back pressure of 0.7 bar was administered to the reactor. Process values were monitored and recorded using the integrated Bioengineering Inc. software (BioSCADA Lab).

#### Saccharification

Saccharification experiments were carried out using deacetylated, dilute acid-pretreated corn stover. The biomass was analyzed for moisture content using an automatic moisture analyzer (Mettler Toledo Moisture Analyzer HB43-S, Mettler Toledo, Greifensee, Switzerland) and carbohydrate composition was determined as previously described [33]. These measurements were used to target a biomass loading of 2% and an enzyme loading of 20 mg/g glucan. Saccharification was performed at two different temperatures (50 and 60 °C) using two different enzyme mixtures: enzymes produced by the fungus *T. aurantiacus* and Novozymes Cellic CTec2. Saccharification reactions (50 mL) were set up in triplicate alongside biomass and enzyme controls using 2% biomass, 100 mM citrate buffer (pH = 5), and 20 mg/g glucan *T. aurantiacus* or CTec2. Reactions were continuously mixed at 180 rpm and 500 µL aliquots were collected every 24 h for 96 h. At each sampling time point, the volume was adjusted for evaporation with distilled H_2_O. Sugar concentrations were measured via HPLC for glucose and xylose and concentrations from the controls were subtracted from their respective samples before calculating yield.

#### Analytical techniques

Quantification of monosaccharides in the hydrolysates was conducted using a high-performance liquid chromatography (Thermo Fisher Scientific, Ultimate 3000, Waltham, MA, USA), which is equipped with a Aminex HPX-87H column (Bio-Rad, 300 × 7.8 mm, Hercules, CA, USA) and a refractive index (RI) detector. The mobile phase is 4 mM H_2_SO_4_ with a flow rate at 0.6 mL/min and column oven temperature at 65 °C. RI detector is heated at 50 °C. The samples were filtered using 0.45 µm centrifuge filters and then diluted with water for injection. Sugar concentrations of the fermentation broth were quantified by high-performance anion-exchange chromatography equipped with a Pulsed Amperometric Detector (ICS-3000 HPAEC-PAD, Dionex, Sunnyvale, CA, USA) with a carbohydrate quadruple waveform due to the low concentrations of the sugars present in the samples. Dionex CarboPac SA10 column was used to separate the sugars at the following conditions: flow rate, 1 mL/min; temperature, 45 °C; eluent, 5 mM NaOH; injection volume, 1 µL.

For SDS-PAGE analysis, gels (8–16% Tris–glycine mini gel; Invitrogen, Carlsbad, CA, USA) were loaded with 20 μL of protein solution [15 μL filtered culture supernatant and 5 μL Laemmli buffer/2-mercaptoethanol (four parts plus one part, respectively)] and 5 µL of Novex sharp prestained protein standard molecular weight markers (Thermo Fisher Scientific, South San Francisco, CA USA). Electrophoresis was carried out at 140 V for 40 min and gels were stained for 1 h using SimplyBlue safe stain (Thermo Fisher Scientific, South San Francisco, CA USA) and destained with distilled, deionized water over night. Total protein concentration of culture supernatants were estimated by the Bradford assay (Bio-Rad, Hercules, CA, USA) in 96-well plates with bovine gamma globulin (0–1 g/L) as standards (Thermo Fisher Scientific, South San Francisco, CA USA). The commonly employed standard, bovine serum albumin (BSA) was not used for protein estimation, because previous reports indicated that it underestimated the protein concentrations in fungal culture broths [34]. The alternative standard, bovine gamma globulin was used, which is less sensitive than the BSA standard and gave results that were more consistent with densitometric analysis of the SDS-PAGE gels [35]. CMCase and xylanase activity measurements were based on quantification of reducing sugars using 3,5-dinitrosalicylic acid (DNS) and OD readings at *λ* = 540 nm. Sugars liberated from sodium carboxymethylcellulose (CMC) or beechwood xylan (Megazyme), were determined using glucose and xylose as standards, respectively. Enzymatic conversion was performed in 96-well plates (80 μL reaction volume) at 65 °C and pH = 5 in 50 mM NaAc for 30 min. 10 μL of diluted culture supernatant (1:50 for CMCase activity and 1:250 for xylanase activity) were used. Enzyme activity assays were carried out in technical triplicates using a liquid handling robotic system (Biomek NX^P^, Beckman Coulter). One unit of CMCase activity (U/mL) was defined as amount of released sugar (nmol) per time (min) per volume of culture supernatant (mL).
